# Urinary stone analysis and clinical characteristics of 496 patients in Taiwan

**DOI:** 10.1038/s41598-024-64869-w

**Published:** 2024-06-19

**Authors:** Wan-Yu Cheng, Jen-Shu Tseng

**Affiliations:** 1 School of Medicine, MacKay Medical College, No. 46, Sec. 3, Zhongzheng Rd., Sanzhi Dist., New Taipei City, 252 Taiwan; 2 Post Graduate Year (PGY) Training, Chi Mei Medical Center, Tainan, Taiwan; 3 Department of Urology, MacKay Memorial Hospital, No.92, Sec. 2, Zhongshan N. Rd., Zhongshan Dist., Taipei City, 104 Taiwan; 4 Institute of Biomedical Informatics, National Yang Ming Chiao Tung University, Taipei, Taiwan

**Keywords:** Epidemiology, Risk factors, Urology, Renal calculi

## Abstract

Evaluate urinary stone components' epidemiological features in urolithiasis individuals and explore potential correlations between stone components and patients' clinical characteristics. A retrospective analysis of urinary stone compositions in 496 patients from a northern Taiwan medical center (February 2006 to October 2021) was conducted. We investigated associations between sex, age, body mass index (BMI), hypertension, diabetes mellitus (DM), hyperlipidemia (HLP), gout, coronary artery disease (CAD), cerebral vascular accident (CVA), chronic kidney disease (CKD), habits, urine pH, and three main stone groups: calcium oxalate (CaOx), calcium phosphate (CaP), and uric acid (UA). Males accounted for 66.5% of cases, with a male-to-female ratio of 1.99:1. Males were negatively associated with CaP stones (OR 0.313, p < 0.001) and positively with UA stones (OR 2.456, p = 0.009). Age showed a negative correlation with CaOx stones (OR 0.987, p = 0.040) and a positive correlation with UA stones (OR 1.023, p < 0.001). DM had a protective effect against CaP stones (OR 0.316, p = 0.004). Gout had a positive association with UA stones (OR 2.085, p = 0.035). Smoking was adversely associated with UA stones (OR 0.350, p = 0.018). Higher urine pH was a risk factor for CaP stones (OR 1.641, p = 0.001) and a protective factor against UA stones (OR 0.296, p < 0.001). These results may provide insights into the pathogenesis of urinary stones and the development of preventative strategies for high-risk populations. Further research is required to confirm and expand upon these findings.

## Introduction

Urolithiasis, a prevalent urologic condition globally, has an Asian incidence of 5 to 19.1%^1^. Despite advancements in clinical diagnoses and treatments, its global incidence and recurrence have remained stable or increased. Given its substantial disease burden, there's a need for international approaches to prevent and manage urolithiasis. In Taiwan, the reported prevalence is 9.01%, with a 5-year recurrence rate of 34.71%^2,3^. Urolithiasis prevalence varies based on factors like age, sex, geography, season, climate, race, obesity, dietary habits, and water intake. While some studies have explored regional stone composition in Taiwan^4,5^, few have investigated the relationship between stone composition and these factors. This study aims to assess changes in stone composition and their association with characteristics.

## Methods

### Study population

We retrospectively reviewed urinary stone compositions in 1039 patients at a northern Taiwan medical center from February 21, 2006, to October 30, 2021. Stones were obtained via spontaneous passage (with or without extracorporeal shock wave lithotripsy in 3 months) or procedures, including open surgery, percutaneous nephrolithotomy, ureteroscopic lithotripsy, endoscopic cystolithotripsy, and so on. Data collected included age, sex, Body Mass Index (BMI), urine pH, and medical history of diabetes mellitus (DM), hypertension (HTN), hyperlipidemia (HLP), gout, coronary artery disease (CAD), cerebral vascular accident (CVA), chronic kidney disease (CKD). Medical history relied on medical records, patient self-report, or documented chronic medication intake. Patient self-reports provided data on alcohol, betel nuts, and cigarette consumption. Stones arising after habit discontinuation were labeled as non-substance users. Missing information led to data exclusion.

### Stone composition grouping

Stone composition analysis employed a Fourier-transform infrared (FTIR) spectrometer. Following the Mayo Clinic classification and European Urological Association guidelines^6,7^, we divided stone composition into five groups: (1) Calcium oxalate (CaOx) group (>50% calcium oxalate monohydrate or dihydrate); (2) Calcium phosphate (CaP) group (>50% tricalcium phosphate, brushite, or carbapatite); (3) Calcium carbonate (CaCO3) group (>50% calcium carbonate); (4) Uric acid (UA) group (>50% uric acid, uric acid dihydrate, or sodium urate); (5) Infection group (>10% struvite or ammonium acid urate); (6) Cystine group (any cystine); (7) Mixed group (CaOx, CaP, and CaCO3, each < 50%).

### Statistical analysis

**Figure 1:**
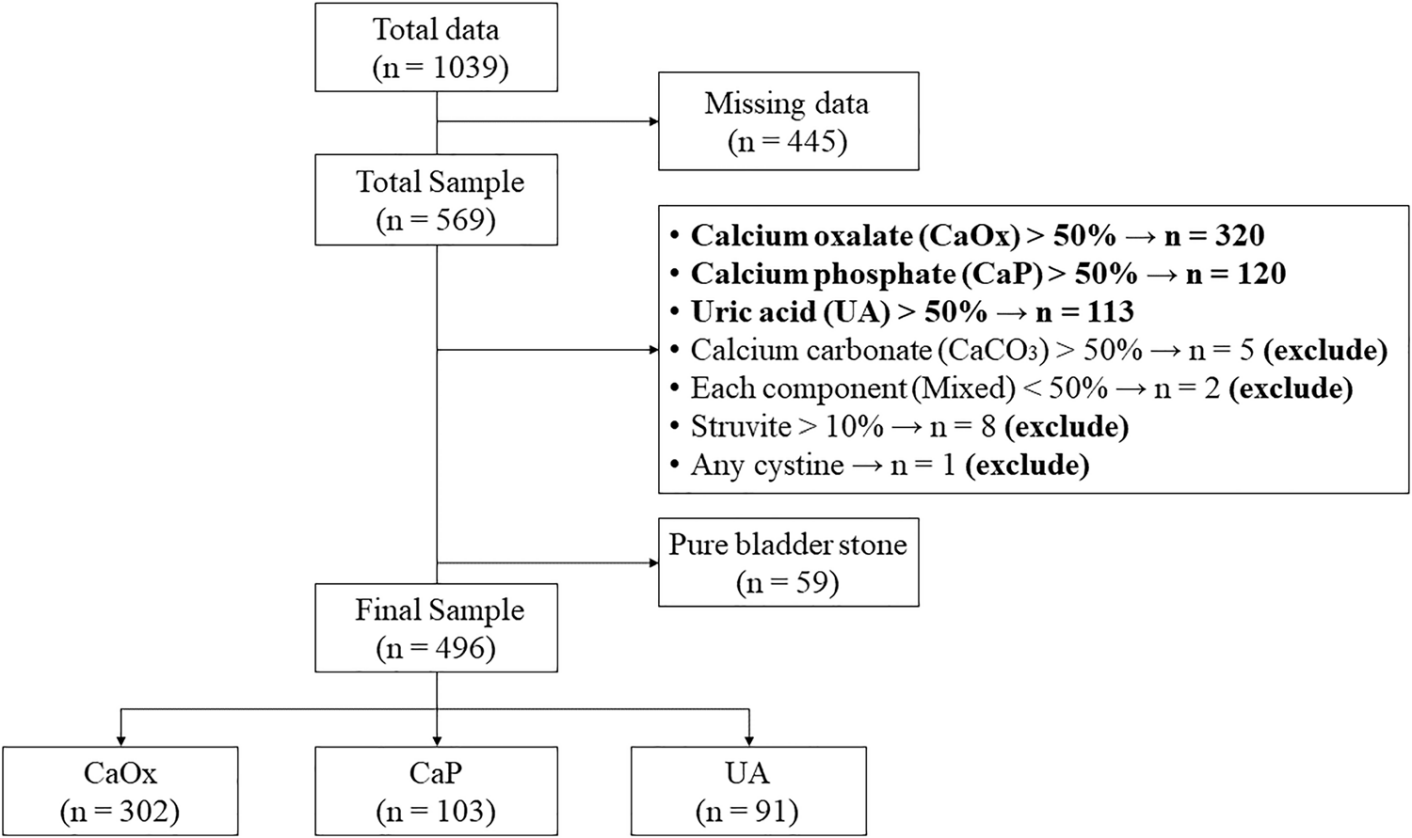
Flowchart of the study design and patient groups.

A total of 569 data entries were included in the analysis, but groups with fewer than 30 observations were excluded to ensure effective statistical analysis. Pure bladder or urethral stones were excluded. Consequently, 496 stones were categorized into three main groups: CaOx, CaP, and UA [Fig. 1]. Univariate and multivariate logistic regression analyses calculated odds ratios (ORs) for the different groups. SAS software 9.4 (SAS Inc., Cary, N.C., USA) was used for all statistical analyses, with a significance level set at p < 0.05.

### Ethics approval

The study protocol was reviewed and approved by the Research Ethics Committee on Human Beings of MacKay Memorial Hospital (Reference: 22MMHIS156e). With institutional IRB approval, informed consent was waived since this study belongs to the lowest risk category, and the potential risks to research subjects do not exceed those of non-participants. Research cannot be conducted without obtaining prior informed consent from research subjects, and this does not affect the rights and interests of research subjects. All experiments performed in studies involving human participants were in accordance with the ethical standards of the institutional research committee and with all guidelines of the Declaration of Helsinki.

## Results

### Sample sources

In this study, we analyzed a total of 569 samples to investigate the prevalence of different sample sources related to kidney stone management. As Table 1 showed, the most common sample source was ureteroscopic lithotripsy, accounting for 48.7% of the total samples. Following closely, spontaneous passage contributed 15.3%, while percutaneous nephrolithotomy represented 16.2% of the samples. Extracorporeal shock wave lithotripsy (ESWL), defined as the expulsion of stones within three months after ESWL treatment, accounted for 4.8%. Most pure lower urinary tract stones were obtained through Cystolithotomy or Endoscopic cystolithotripsy, with only 2 cases being spontaneously passed.

**Table 1:** The distribution of sample sources for stone analysis.

Sample source	N (%)
Spontaneous passage	87 (15.3)
Extracorporeal shock wave lithotripsy	27 (4.8)
Ureteroscopic lithotripsy	277 (48.7)
Retrograde intrarenal surgery	27 (4.8)
Laparoscopic nephrectomy	1 (0.2)
Open nephrolithotomy	1 (0.2)
Percutaneous nephrolithotomy	92 (16.2)
Cystolithotomy	5 (0.9)
Endoscopic cystolithotripsy	52 (9.1)
Total	569 (100)

### Stone composition

**Figure 2:**
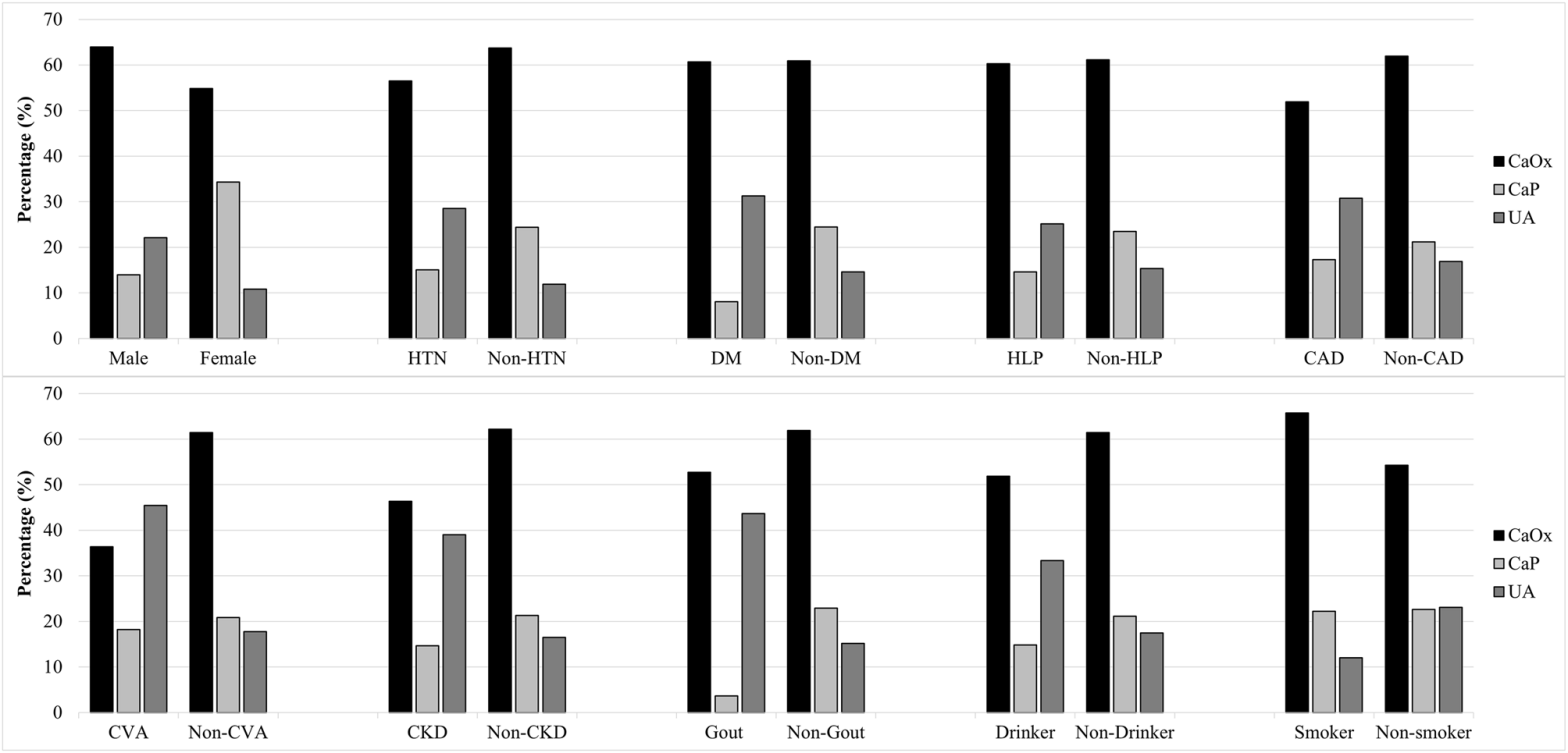
Percentage of stone composition by different characteristics. *CaOx* Calcium oxalate, *CaP* Calcium phosphate, *UA* Uric acid, *DM* Diabetes Mellitus, *HTN* Hypertension, *HLP* Hyperlipidemia, *CAD* Coronary artery disease, *CVA* Cerebral vascular accident, *CKD* Chronic kidney disease.

As Table 2 showed, CaOx was the most prevalent stone component, constituting 320 (56.2) stones. Followed by 120 (21.1%) stones of CaP, and 113 (19.9%) stones of UA. Other stone components included 5 (0.9%) stones of CaCO3, 8 (1.4%) stones of struvite, 1 (0.2%) stone of cystine, and 2 (0.4%) stones of mixed component. The distribution of stone composition based on various characteristics is illustrated in Fig. 2. After excluding the 59 cases of pure lower urinary tract stones, which account for approximately 10%, with the most prevalent component being UA, the distribution of upper urinary tract stone components follows a similar order to the overall stone component distribution.

**Table 2:** Distribution of main stone composition.

Composition group	Lower (%)	Upper (%)	Total (%)
Calcium oxalate	18 (30.5)	302 (59.2)	320 (56.2)
Calcium phosphate	17 (28.8)	103 (20.2)	120 (21.1)
Uric acid	22 (37.3)	91 (17.8)	113 (19.9)
Calcium carbonate	0 (0)	5 (1)	5 (0.9)
Infection	1 (1.7)	7 (1.4)	8 (1.4)
Cystine	0 (0)	1 (0.2)	1 (0.2)
Mixed	1 (1.7)	1 (0.2)	2 (0.4)
Total	59 (10.4)	510 (89.6)	569 (100)

### Sex

In our investigation, 330 (66.5%) of the cases were male, making the male-to-female ratio 1.99: 1. CaOx stones were predominant in both sexes [Table 3]. In univariate analysis, significant correlations were identified between sex and the different types of stones, including CaOx (p = 0.05), CaP (p < 0.001), and UA (p = 0.003) stones. In multivariate analysis, sex and CaOx did not show significant differences (p = 0.099). Additionally, male exhibited a significant positive association with an increased likelihood of UA stones (OR 2.456, p = 0.009). However, it revealed that male demonstrated a significant inverse association with CaP stones (OR 0.313, p < 0.001) [Table 4].

**Table 3:** Comparison of stone components in patients with different clinical characteristics.

Index	CaOx	CaP	UA	N (%)
Overall (%)	302 (60.9)	103 (20.8)	91 (18.3)	496 (100)
Sex (%)				
Male	211 (64.0)	46 (13.9)	73 (22.1)	330 (66.5)
Female	91 (54.9)	57 (34.3)	18 (10.8)	166 (33.5)
Age (%)				
0 ~ 18	4 (50.0)	1 (12.5)	3 (37.5)	8 (1.6)
19 ~ 40	69 (65.7)	26 (24.8)	10 (9.5)	105 (21.2)
41 ~ 65	181 (61.6)	63 (21.4)	50 (17.0)	294 (59.3)
66 ~ 100	48 (53.9)	13 (14.6)	28 (31.5)	89 (17.9)
Hypertension (%)				
Yes	109 (56.5)	29 (15.0)	55 (28.5)	193 (38.9)
No	193 (63.7)	74 (24.4)	36 (11.9)	303 (61.1)
Diabetes mellitus (%)				
Yes	68 (60.7)	9 (8.0)	35 (31.3)	112 (22.6)
No	234 (60.9)	94 (24.5)	56 (14.6)	384 (77.4)
Hyperlipidemia (%)				
Yes	91 (60.2)	22 (14.6)	38 (25.2)	151 (30.4)
No	211 (61.2)	81 (23.5)	53 (15.3)	345 (69.6)
Coronary artery disease (%)				
Yes	27 (51.9)	9 (17.3)	16 (30.8)	52 (10.5)
No	275 (61.9)	94 (21.2)	75 (16.9)	444 (89.5)
Cerebral vascular accident (%)				
Yes	4 (36.4)	2 (18.2)	5 (45.4)	11 (2.2)
No	298 (61.5)	101 (20.8)	86 (17.7)	485 (97.8)
Chronic kidney disease (%)				
Yes	19 (46.4)	6 (14.6)	16 (39.0)	41 (8.3)
No	283 (62.2)	97 (21.3)	75 (16.5)	455 (91.7)
Gout (%)				
Yes	29 (52.7)	2 (3.7)	24 (43.6)	55 (11.1)
No	273 (61.9)	101 (22.9)	67 (15.2)	441 (88.9)
Smoking (%)				
Yes	64 (67.4)	21 (22.1)	10 (10.5)	95 (19.2)
No	238 (59.3)	82 (20.5)	81 (20.2)	401 (80.8)
Alcohol (%)				
Yes	14 (51.9)	4 (14.8)	9 (33.3)	27 (5.4)
No	288 (61.4)	99 (21.1)	82 (17.5)	469 (94.6)
Betel nut (%)				
Yes	5 (83.3)	1 (16.7)	0 (0)	6 (1.2)
No	297 (60.6)	102 (20.8)	91 (18.6)	490 (98.8)
Urine pH (%)				
≥ 7	69 (68.3)	28 (27.7)	4 (4.0)	101 (20.4)
< 7	233 (59.0)	75 (19.0)	87 (22.0)	395 (79.6)

*CaOx* Calcium oxalate, *CaP* Calcium phosphate, *UA* Uric acid.

**Table 4:** Comparative logistics regression analysis of stone composition.

Variable	CaO_X_				CaP				UA			
	Univariate		Multivariate		Univariate		Multivariate		Univariate		Multivariate	
	OR (95% CI)	p-value	OR (95% CI)	p-value	OR (95% CI)	p-value	OR (95% CI)	p-value	OR (95% CI)	p-value	OR (95% CI)	p-value
Sex (Male)	1.461 (1.000; 2.136)	0.05*	1.383 (0.940; 2.035)	0.099	0.310 (0.198; 0.484)	<0.001**	0.313 (0.194; 0.505)	<0.001**	2.335 (1.342; 4.064)	0.003*	2.456 (1.253; 4.811)	0.009*
Age	0.984 (0.972; 0.996)	0.009*	0.987 (0.974; 0.999)	0.040*	0.992 (0.978; 1.006)	0.277			1.038 (1.021; 1.056)	<0.001**	1.023 (1.000; 1.046)	0.046*
BMI	0.967 (0.929; 1.007)	0.108			0.991 (0.944; 1.041)	0.716			1.062 (1.011; 1.115)	0.017*	1.024 (0.961; 1.091)	0.466
Hypertension	0.740 (0.512; 1.069)	0.109			0.547 (0.341; 0.879)	0.013*	0.752 (0.436; 1.297)	0.306	2.956 (1.852; 4.719)	<0.001**	1.588 (0.862; 2.925)	0.138
Diabetes mellitus	0.991 (0.644; 1.525)	0.966			0.270 (0.131; 0.554)	<0.001**	0.316 (0.144; 0.694)	0.004*	2.662 (1.631; 4.345)	<0.001**	1.338 (0.723; 2.475)	0.353
Hyperlipidemia	0.963 (0.651; 1.424)	0.851			0.556 (0.332; 0.931)	0.026*	0.844 (0.467; 1.525)	0.573	1.853 (1.158; 2.964)	0.010*	1.075 (0.594; 1.945)	0.811
CAD	0.664 (0.373; 1.182)	0.164			0.779 (0.367; 1.656)	0.517			2.187 (1.154; 4.143)	0.016*	1.064 (0.482; 2.345)	0.879
CVA	0.359 (0.104; 1.242)	0.106			0.845 (0.180; 3.972)	0.831			3.866 (1.154; 12.959)	0.028*	1.623 (0.382; 6.891)	0.512
CKD	0.525 (0.276; 0.998)	0.049*	0.597 (0.309; 1.153)	0.124	0.633 (0.259; 1.548)	0.316			3.243 (1.652; 6.366)	<0.001**	1.730 (0.765; 3.914)	0.189
Gout	0.686 (0.391; 1.205)	0.190			0.127 (0.030; 0.530)	0.005*	0.263 (0.061; 1.145)	0.075	4.322 (2.389; 7.819)	<0.001**	2.085 (1.055; 4.120)	0.035*
Smoking	1.414 (0.881; 2.269)	0.151			1.104 (0.642; 1.898)	0.721			0.465 (0.231; 0.935)	0.032*	0.350 (0.147; 0.836)	0.018*
Alcohol	0.677 (0.311; 1.473)	0.325			0.651 (0.220; 1.924)	0.437			2.360 (1.024; 5.438)	0.044*	2.649 (0.941; 7.454)	0.065
Betel nut	3.249 (0.377; 28.020)	0.284			0.761 (0.088; 6.584)	0.804						
Urine pH	1.239 (0.977; 1.571)	0.077			1.988 (1.500; 2.634)	<0.001**	1.641 (1.217; 2.214)	0.001*	0.768 (0.739; 0.799)	<0.001**	0.296 (0.187; 0.470)	<0.001**

*CaOx* Calcium oxalate, *CaP* Calcium phosphate, *UA* Uric acid, *CAD* Coronary artery disease, *CVA* Cerebral vascular accident, *CKD* Chronic kidney disease.

* < 0.05, ** < 0.001.

### Age

**Figure 3:**
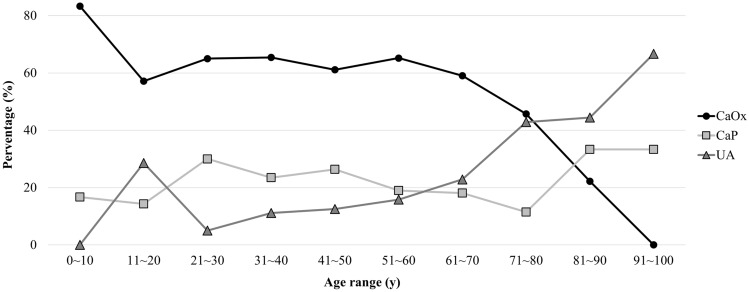
Percentage of stone composition by age group. *CaOx* Calcium oxalate, *CaP* Calcium phosphate, *UA* Uric acid.

The age of the patients ranged from 0 to 100, with an average age of 52.9 ± 15.2 years. 8 (1.6%) stones were from individuals aged 0–18, 105 (21.2%) from those aged 19–40, 294 (59.3%) from patients aged 41–65, and 89 (17.9%) from patients aged 66–100. CaOx remained the predominant substance in each age group [Table 3]. Significantly, the trend of stone composition underwent noteworthy changes with increasing age [Fig. 3]. In univariate analysis, sex showed significant correlations with different stone types, including CaOx (p = 0.009) and UA (p < 0.001) stones. In multivariate analysis, age retained its negative association, indicating a protective effect against CaOx stones (OR 0.987, p = 0.040). Conversely, for UA stones, a significant positive association was observed with increasing age (OR 1.023, p < 0.001) [Table 4].

### BMI

Significant relationships were discovered in the univariate analysis between BMI and UA (p = 0.017) stones. However, it didn’t retain its significant positive correlation with UA stones (p = 0.466) in the multivariate analysis. And there was no significant relationship between BMI and the CaOx or CaP stone groups [Table 4].

### Underlying diseases

Among the diseases DM, HTN, HLP, gout, CAD, CVA, and CKD, CaOx showed a significant association solely with CKD (OR 0.525, p = 0.049) in univariate regression, while no significant associations were observed with other diseases and multivariate analysis. CaP exhibited significant negative associations with DM, HTN, HLP, and gout in univariate regression analysis. However, in multivariate regression analysis, only DM (OR 0.316, p = 0.004) maintained their significance. UA displayed significant positive associations with all diseases in univariate regression, but only gout maintained its significance in multivariate regression (OR 2.085, p = 0.035). [Table 4].

### Habits

Smoking only had a significant negative association with UA stones (OR 0.465, p = 0.032) in univariate analysis. This correlation persisted in multivariate analysis of UA stones (OR 0.350, p = 0.018), indicating that smoking was a protective factor against the formation of UA stones [Table 4]. While alcohol exhibited no correlation with any stone composition in both the univariate and multivariate analyses.

### Urine pH

Urine pH had no significant relationships with CaOx stones in both regression study. In univariate regression analysis, urine pH exhibited a significant positive association with CaP stones (OR 1.988, p < 0.001), while UA stones showed a significant negative association (OR 0.768, p < 0.001). This association persisted in multivariate analysis for both CaP (OR 1.641, p = 0.001) and UA stones (OR 0.296, p < 0.001), indicating that alkaline urine is a risk factor for CaP stones and a protective factor against UA stone development [Table 4].

## Discussion

The incidence of bladder calculi has dramatically declined in recent years along with economic growth. Bladder calculi make up between 3% and 15% of all urinary stones in Asia^8^. Similar findings were found in our study, where bladder calculi accounted for about 10.4% of cases. Our study also revealed that CaOx stones were more frequently found in the upper urinary tract, while pure lower urinary tract stones were predominantly identified as UA stones. Additionally, the Chi-Square test demonstrated a significant difference between stone location and composition. This suggests that the etiologies of upper and lower urinary tract stones may vary, which was compatible with the previous study^9^. Consequently, we excluded pure lower urinary tract stones in this study.

Consistent with other studies, our research [Table 2] also identified CaOx as the most prevalent stone component, with a lower proportion compared to other Asian countries, while CaP had a relatively higher proportion. A research in an Asian population^8^ found that CaOx accounted for 75-90% of upper urinary tract stones, followed by UA (5-20%), CaP (6-13%), struvite (2-15%), apatite (1%), and cystine (0.5-1%).

In this study, a noticeable sex disparity was observed, with a male-to-female composition ratio of 1.99:1 [Table 2]. This finding is consistent with previous research conducted in Taiwan^10^, China^11^, and other Asian countries^8^. The higher occurrence of CaOx and UA stones in males may be attributed to the higher levels of androgens^12^ and lower urine pH, which have been shown to promote their formation. In the CaP groups, females were found to have a higher proportion than males. This could be attributed to the anatomical characteristics of females, which may make them more prone to urinary tract infections^13^, and be associated with alkaline urine.

Based on this study, urolithiasis is exceptionally rare in individuals below the age of 18 and beyond the age of 80. After reaching 18 years of age, the prevalence of urolithiasis steadily rises, peaking at 51–60-year-old middle-aged individuals, and then steadily declines, which is consistent with trends observed in most countries. As the predominant workforce in society, middle-aged individuals frequently participate in strenuous occupations, which can lead to unhealthy lifestyles characterized by irregular eating and sleeping patterns, insufficient fluid intake, sedentary behavior, and chronic occupational stress. The cumulative effect of these factors may elevate the risk of stone formation in middle-aged individuals. Consistent with the results in previous studies^14^, there is a trend of decreased proportion of CaOx stones with ageing. This trend may be associated with a decrease in urinary calcium excretion as individuals age^15^. Conversely, as patients aged, the proportion of UA stones rose, which was compatible with previous studies^16^. This could be related to the decline in renal function, urine pH and increased UA excretion in the elderly.

BMI is an indicator of obesity and is positively correlated with the risk of kidney stones, particularly in the production of UA and CaOx stones^17^. Adipose tissue in obese individuals releases pro-inflammatory and oxidative cytokines, exacerbating the relationship between obesity and kidney stone production by causing renal tissue damage and crystal deposition. UA stones are associated with factors like insulin resistance, renal fat deposition, and ammonia excretion defects. Dietary habits prevalent in obesity, such as high meat consumption, contribute to increased purine load and a reduction in urine pH, further elevating the risk of UA stone formation. Additionally, obesity elevates the risk of CaOx stones through heightened endogenous oxalate synthesis, increased intestinal absorption of oxalate, and elevated urinary oxalate excretion^18^. However, in our study, the findings for CaOx and UA stones showed no significant correlation, even when patients with a BMI > 25 were categorized as obese.

Hypertension is linked to increased calcium and oxalate excretion, heightened UA excretion in men, reduced citrate excretion, lower urine pH, and increased titratable acid excretion, contributing to a higher oversaturation of CaOx^19^. Several previous studies have found a higher incidence of UA stones among individuals with hypertension.

Type 2 DM has been recognized as a significant risk factor for kidney stone development, particularly UA stones^16^. Insulin resistance-induced hyperglycemia leads to the formation of advanced glycation end products (AGEs), triggering a pro-inflammatory response and causing vascular endothelial damage^20^. This condition not only decreases ammonium synthesis, leading to lower urine pH and an increased risk of UA stone formation but also elevates the risk of calcium stone formation by reducing urine citrate excretion^21^.

Dyslipidemia is associated with an elevated risk of kidney stones through various pathways, although the specific mechanisms contributing to this heightened risk remain undefined. Reduced levels of High-density lipoprotein (HDL) and elevated triglyceride (TG) levels, indicative of insufficient exercise and insulin resistance, are correlated with increased excretion of urinary sodium, oxalate, and UA, as well as a decrease in urine pH. These factors collectively elevate the likelihood of UA stone formation^22^. Additionally, heightened total cholesterol levels are significantly associated with increased urinary potassium and calcium excretion, potentially raising the risk of calcium stone formation.

Similarly to a previous study^23^, no significant relationship was found between the CaOx component and metabolic syndrome factors, even though these factors have been demonstrated to impact urinary levels of calcium, oxalate, and citrate. HTN, DM, and HLP were significantly linked to CaP and UA components in univariate analysis. Nevertheless, in multivariate analysis, only the association between CaP stones and DM remained negative. The aforementioned information confirms that metabolic syndrome factors as well as gout are correlated with low urinary pH, influencing the crystallization of both stone compositions, and resulting in differences in their composition.

Gout is a recognized risk factor for the formation of urinary stones^24^, particularly CaOx and UA stones. In patients with gout, uric acid stones are associated with a lower urinary pH, hyperuricemia and reduced fractional excretion of uric acid^25^. Moreover, inflammatory conditions and associated metabolic complications may elevate the risk of nephrolithiasis in patients with gout^26^. Aligning with prior study^27^, our research also indicated a significant positive relationship between gout and uric acid stones. Reducing urinary uric acid excretion with allopurinol has been shown to decrease the risk of CaOx stone formation^28^, and it alters the stone composition distribution in gout patients to resemble that of patients without gout. The lack of documented medication history for patients in our study might have influenced the absence of notable differences between the two groups.

Nephrolithiasis increases the risk of cardiovascular illnesses, especially coronary heart disease and stroke^29^, by being associated with atherosclerosis and vascular calcification. According to Ferraro et al.^30^, there is a connection between an increased risk of cardiovascular disease (CVD) and the concentration of CaP in stones. Bargagli et al.^31^ found that individuals with comorbid kidney stones and CVD tend to have reduced urinary excretion of citrate and magnesium, as well as a lower urine pH. Low urine citrate levels are frequently seen in those who develop CaP stones, and magnesium is essential for cardiovascular health and for preventing CaOx crystallization. Nevertheless, our study was consisted with earlier research, which did not discover a connection between stone composition and CVD^31,32^.

Studies conducted in Taiwan^33^ and the US^34^ showed that individuals with UA stones had much worse renal function than patients with CaP stones. Furthermore, patients with a lower estimated glomerular filtration rate (eGFR) exhibited a statistically significant correlation with reduced urinary pH, and a diminished urinary excretion of calcium and citrate. Li et al.^35^ also found a higher prevalence of CKD among patients with UA stones. It may be necessary to closely monitor renal function in patients with UA stones during follow-up. Our research indicated that UA stones have a significant positive correlation with CKD only in univariate regression analysis. The lack of detailed recording or documentation regarding CKD stages and biochemical indicators of renal function may have contributed to the observed differences in findings.

Prior research in southern Taiwan^36^ found that calcium urolithiasis development was independently influenced by current cigarette smoking, betel chewing, but not alcohol consumption. Smoking has been linked to an increased risk of urolithiasis due to its high concentration of toxic chemicals. Tobacco smoke has been found to elevate blood and kidney lead and cadmium levels, showing a positive association with CaOx stones and a negative association with UA stones^37^. It may also elevate vasopressin levels, exerting a strong vasoconstrictive effect, reducing urine output, and increasing the risk of urolithiasis. Additionally, it has been shown to be a separate risk factor for calcium urolithiasis, potentially contributing to the increased accumulation of calcium in the kidneys or reduced elimination of calcium through urine^38^. Smoking's potential to release reactive oxygen species (ROS) may induce kidney damage and hasten the progression of chronic kidney disease, an acknowledged risk factor for lithogenesis. Moreover, smokers exhibit a lower incidence of UA stones compared to non-smokers, possibly attributed to reduced endogenous UA levels^39^, which may be linked to the antioxidant effect on ROS and free radicals produced by cigarettes and the depletion of antioxidants^40^. The precise effect of smoking on UA levels is still being debated. However, our study did not include the concentration of serum UA, so this aspect remains unknown. Despite no measurement of cadmium or lead concentrations, our study revealed that smoking negatively correlated with UA stones.

Consistent with our findings, urine pH significantly influences kidney stone formation. Most research indicates CaOx stones can form at any pH, with a consensus that their supersaturation is pH-independent. Conversely, CaP supersaturation rises rapidly with a urine pH above 6, particularly in the presence of hypercalciuria and hypocitraturia in alkaline urine^41^. As mentioned, UA stone formation might be attributed to aciduria, hyperuricosuria, or hyperuricemia, which could be caused by insulin resistance, altered purine metabolism, or impaired renal function. Among these factors, low urinary pH has been identified as having the most significant impact, resulting in reduced solubility of UA^42^. Decreased urine pH has been confirmed to be associated with BMI, metabolic factors, and HOMA-IR, which is a measure of insulin resistance.

Regarding study limitations, it is important to acknowledge that our stone analysis data were sourced from samples obtained during natural passage or surgery. The decision to submit samples for analysis relied on the discretion of the treating physician, lacking a standardized screening protocol, which could introduce bias. Additionally, the study's reliance on data from a single medical center and the relatively small sample size may limit the generalizability of the findings to the broader population. Finally, the retrospective design could introduce data inaccuracies, and we did not collect comprehensive biochemical data such as serum calcium, phosphate, UA levels, or 24-hour urine analyses, as well as information on the history of medication use, which could influence stone formation. Despite its limitations, the study provides insights into urolithiasis characteristics, highlighting the need for larger, multicenter research to comprehensively understand the topic, possibly utilizing Taiwan’s National Health Insurance Research Database for diverse regional insights.

## Conclusion

In conclusion, the results show significant associations between the composition of stones and variables such sex, age, BMI, metabolic diseases, gout, smoking habits, and urine pH. The most prevalent stone type was found to be CaOx, with men showing a higher prevalence to UA stones and women to CaP stones. Age exhibits a negative correlation with the occurrence of CaOx stones and a positive correlation with UA stones. Among metabolic diseases, while only DM displayed a significant inverse association with CaP stones, and gout showed a strong association with UA stones. Additionally, smokers had a lower risk of UA stones. Finally, CaP stones are associated with alkaline urine, whereas UA stones are linked to acidic urine conditions. These findings may offer valuable information for understanding urolithiasis pathogenesis and developing preventative strategies for high-risk populations. Further research is warranted to validate and expand upon these findings.

## Data Availability

The data that support the findings of this study are available on request from the corresponding author upon reasonable request.

## Abbreviations

BMI: Body mass index
CAD: Coronary artery disease
CaCO3: Calcium carbonate
CaOx: Calcium oxalate
CaP: Calcium phosphate
CKD: Chronic kidney disease
CVA: Cerebral vascular accident
CVD: Cardiovascular disease
DM: Diabetes mellitus
HLP: Hyperlipidemia
HTN: Hypertension
UA: Uric acid
